# Molecular detection of a novel cyprinid herpesvirus in roach (*Rutilus rutilus*) and asp (*Leuciscus aspius*) showing typical signs of carp pox disease

**DOI:** 10.1007/s00705-020-04638-y

**Published:** 2020-05-01

**Authors:** Boglárka Sellyei, Ferenc Baska, Ádám Varga, Réka Borzák, Andor Doszpoly

**Affiliations:** 1 Institute for Veterinary Medical Research, Centre for Agricultural Research, Hungarian Academy of Sciences, P.O. Box 18, Budapest, 1581 Hungary; 2 Department of Exotic Animal and Wildlife Medicine, University of Veterinary Medicine, Budapest, Hungary

## Abstract

In the early spring of 2018, in Lake Balaton (Hungary), a roach (*Rutilus rutilus*) and an asp (*Leuciscus aspius*) were found in an fish trap at the outlet of the river Sió showing typical signs of the so-called carp pox disease, such as foci of epidermal hyperplasia on the head and the whole body surface, including the fins. Molecular tests revealed the presence of the DNA of an unknown fish herpesvirus. Three genes encoding the DNA-dependent DNA polymerase, major capsid protein and ATPase subunit of terminase were amplified and sequenced from the alloherpesviral genome. The gene sequences of the viruses obtained from the two different fish species shared 94.4% nucleotide sequence identity (98.1% amino acid sequence identity), suggesting that they belong to the same virus species. Phylogenetic analysis based on the DNA polymerase (and the concatenated sequences of the amplified genes, as well) implied that the detected virus belongs to the genus *Cyprinivirus* within the family *Alloherpesviridae*. The sequences of the novel alloherpesvirus diverge from those of the five cyprinivirus species described previously, so it putatively represents the sixth virus species in the genus.

## Introduction

Cyprinid herpesviruses (CyHVs) belong to the family *Alloherpesviridae*, which includes all herpesviruses detected in and/or isolated from amphibian and fish species [6]. The family comprises four genera. The genus *Batrachovirus* includes viruses detected in amphibians, while the viruses of salmonid fish species belong to the genus *Salmonivirus*. Viruses of the genus *Ictalurivirus*, however, were isolated from evolutionary distinct fish species, namely the catfishes and sturgeons. The fourth genus, *Cyprinivirus*, comprises four virus species accepted by the International Committee on Taxonomy of Viruses (ICTV) [26], three of which (*Cyprinid herpesvirus 1*, *Cyprinid herpesvirus 2*, and *Cyprinid herpesvirus 3*) are associated with common carp (*Cyprinus carpio*) or goldfish (*Carassius auratus*) (family Cyprinidae in the order Cypriniformes) and one of which (*Anguillid herpesvirus 1*) is associated with European eel (*Anguilla anguilla*) (family Anguillidae in the order Anguilliformes). Diseases caused by fish herpesviruses are common worldwide, and they vary in both severity and signs [28]. Some of them are highly virulent, causing acute systemic infections and high mortality (e.g., ictalurid herpesvirus 1 and 2; cyprinid herpesvirus 2) [1, 14, 21]. Others are also highly virulent, causing acute diseases with high mortality producing integumentary lesions (acipenserid herpesvirus 1 and 2; cyprinid herpesvirus 3) [18, 19, 31]. A subset of the fish herpesviruses are weakly virulent, causing chronic systemic infections (salmonid herpesvirus 1) [11] or mild integumentary lesions (salmonid herpesvirus 4; northern pike herpesvirus; sheatfish herpesvirus) [4, 9, 32].

Cyprinid herpesvirus 1 (CyHV-1) is known to cause epidermal hyperplasia, the so-called carp pox disease. The virus was isolated from common carp more than 30 years ago [27]. Similar signs have been observed – white-to-grey mucoid-to-waxy epidermal growths covering the body surface including the head and fins – in other cyprinid fish species, including barbel (*Barbus barbus*), bleak (*Alburnus alburnus*), bream (*Abramis brama*), chub *(Squalius cephalus*), crucian carp (*Carassius carassius*), orfe (*Leuciscus idus*), roach (*Rutilus rutilus*) and tench (*Tinca tinca*) [7, 22, 24, 30]. However, viruses have not been isolated from those fish species, nor has the etiology been ascertained by molecular methods as being CyHV-1.

Cyprinid herpesvirus 2 (CyHV-2), also known as haematopoietic necrosis virus, was isolated in Japan from goldfish [21], and has been reported in Prussian carp (*Carassius gibelio*) [8]. The affected fish do not show characteristic external signs except for apathy and pale gills. Histopathological findings may typically include mild-to-severe multifocal or diffuse coagulative necrosis in kidney and spleen.

The well-known koi herpesvirus (cyprinid herpesvirus 3 [CyHV-3]), which causes devastating losses in aquaculture worldwide, has been isolated from both common and koi carp [19]. On moribund fish, the most consistent microscopic lesions are seen in the gills, with hyperplasia and hypertrophy of the branchial epithelium and fusion of the secondary lamellae [19].

Few years ago, a novel cyprinid herpesvirus was reported from sichel (*Pelecus cultratus*) and was tentatively named "cyprinid herpesvirus 4" (CyHV-4). In the studied fish, microscopic lesions were observed in the epithelium of renal tubules, as well as congestion and multifocal vacuolisation in the brain stem and cerebellum [10].

The present study was aimed at genetically characterizing a novel alloherpesvirus (AlloHV) detected in roach and asp (*Leuciscus aspius*) showing typical signs of carp pox disease.

## Materials and methods

### Specimen collection

A roach with typical lesions of carp pox disease (25 cm, approx. 0.15 kg) and the carcass of an asp also showing similar external signs (40 cm, approx. 1 kg) were delivered to our laboratory for histopathological and molecular examination. The water temperature of the lake was 12 °C at the time of the collection of the specimens.

After euthanasia, the fish were necropsied, and tissue samples were collected from the main organs of the roach (lesions of skin, gills, brain, liver, kidney, and spleen) for virus isolation and molecular and histopathological examination. Excised tissues were fixed in Bouin’s fixative, washed in 80% ethanol, embedded in paraffin, sectioned (at 4─5 µm), stained with hematoxylin and eosin, and viewed by light microscopy according to standard procedures. Internal organs and hyperplastic tissues were collected from the carcass of the asp for PCR.

### Virus isolation

Virus isolation was attempted on EPC (epithelioma papulosum cyprinid) and KF-1 (caudal fin of koi carp) cell lines [13, 19]. Both cell lines were cultured in MEM medium (Biosera, France) supplemented with 2 mM HEPES buffer (Biosera, France), 10% fetal bovine serum (Biosera, France) and 1% penicillin-streptomycin (Biosera, France) at 25 °C without CO_2_. The homogenates of pooled internal organs and those of the hyperplastic tissues were diluted to a 10% (w/v) suspension in MEM medium (Biosera, France) complemented with antibiotics (penicillin, 300 U/ml; streptomycin, 300 µg/ml). The suspensions were centrifuged at 2000 × g for 10 min at 16 °C. EPC and KF-1 monolayers, 80% confluent, in 25-cm^2^ flasks were inoculated with 1 ml supernatant of sedimented homogenates per flask. The flasks were incubated at 20 °C and 25 °C and checked daily for a cytopathic effect (CPE).

### PCR assays

For molecular investigations, organ samples were homogenized using a TissueLyser high-throughput disruption instrument (QIAGEN, Germany) according to the manufacturer's recommendations. DNA extraction was carried out using a NucleoSpin Tissue Kit (Macherey-Nagel, Germany). Subsequently, the roach and asp tissue samples were tested for the presence of herpesviral DNA, employing widely used PCR methods for the detection of cyprinid herpesviruses targeting the DNA-dependent DNA polymerase (DNA pol) and major capsid protein (MCP) genes (Table 1) [12].

**Table 1:** Primers used for PCR to amplify short regions of the DNA polymerase, major capsid protein, terminase, and helicase genes

Target	Primers	Reference
DNA polymerase	Outer forward: 5’-CCA GCA ACA TGT GCG ACG G-3’ Inner forward: 5’-CGA CGG VGG YAT CAG CCC-3’ Inner reverse: 5’-GAG TTG GCG CAY ACY TTC ATC-3’ Outer reverse: 5’-CCG TAR TGA GAG TTG GCG CA-3’	[12]
Major capsid protein	Outer forward: 5’-CAG ACC AAG AAC TAC GTG GG-3’ Inner forward: 5’-GTC TAY GAC CAG ATG ACC ATG-3’ Reverse: 5’-GCT CAS CAM CGC GGT GTG-3’	[12]
DNA polymerase	Forward: 5’-GGN GCN ATG GTN CAR WSN ACN AA-3’ Reverse: 5’-ACN GTN GCN GTR TTY TCR TAN GC-3’	[10]
Terminase	Forward: 5’-GCG CTG AGK ATG TCG TCY TTG-3’ Reverse: 5’-YGA CAT CTA CAA GCC CGA CCA-3’	[10]
Helicase	Forward: 5’-GTN GGN WSN GTN ACN CAR YT-3’ Reverse: 5’-CCY TGR CAR AAR TAN GTR TTC AT-3’	[10]

For amplifying and sequencing longer regions of the DNA polymerase, ATPase subunit of terminase (terminase), and helicase genes, previously described consensus primers designed for detection of cypriniviruses were used (Table 1) [10]. The PCR mixtures for amplifying the above-mentioned regions contained 34 µl of distilled water, 10 µl of Phusion® 5X Green HF buffer (Thermo Scientific, USA), 1.5 µl of dNTP solution (10 mM), 1 µl of each primer (10 pM), 0.5 µl of Phusion® High-Fidelity DNA polymerase enzyme (Thermo Scientific, USA), and 2 µl of target DNA. The PCR profiles consisted of an initial step at 98 °C for 3 min, followed by 45 cycles of denaturation at 98 °C for 10 s, annealing at 56 °C for 30 s, and elongation at 72 °C for 1 min, and a final extension at 72 °C for 3 min. PCR products were separated in an agarose gel (1%) and visualized under UV light. The DNA fragments were excised and purified using a NucleoSpin Gel and PCR Clean-up Kit (Macherey-Nagel, Germany), and sequenced bi-directionally. The sequencing reactions were performed using a BigDye Terminator v3.1 Cycle Sequencing Kit (Applied Biosystems, USA), and electrophoresis was carried out by a commercial service provider on an ABI PRISM 3100 Genetic Analyzer.

### Phylogenetic analysis

Sequence analysis and contig assembly were carried out using the BioEdit [17] and Staden [29] program packages. The primer sequences were removed from both ends of the sequences of the DNA fragments, and their identity was confirmed using the BLASTx program [2] at NCBI on-line. Phylogenetic relationships within the family *Alloherpesviridae* were inferred based on an alignment of a 79-aa portion of the deduced amino acid sequence of the DNA pol gene from all available AlloHVs. An additional phylogenetic analysis was carried out based on the alignment of 411-aa-long sequences consisting of the concatenated sequences of the DNA polymerase and terminase of the members of the genus *Cyprinivirus*. Mitochondrial sequences (1138 nucleotides [nt] from the cytochrome b gene) were used for phylogenetic identification of the host species. For the multiple alignment, the online Mafft version 7 [23] was used with default parameters. After removal of the gaps, Bayesian phylogenetic analysis was performed using MrBayes [20] in the TOPALi v2.5 program package and interface [25] with the following parameters: default prior probability distribution, Markov chain for 10 million generations, four independent analyses, each with one cold and three heated chains. Sampling was done every 10 generations, with the first 25% of Markov chain Monte Carlo samples discarded as burn-in. A convergence diagnostic test [15] was carried out using the TOPALi v2.5 program. The WAG and JTT amino acid substitution models were found to be the best fit for the DNA polymerase gene and the concatenated sequences, respectively, while for the identification of the host species, the HKY nucleotide substitution model was used. The maximum-likelihood method (Phyml) was also used for phylogenetic analysis with the RTRev (DNA pol) and JTT (concatenated sequences) amino acid substitution models and with the HKY nucleotide substitution model for the mitochondrial sequences. Phyml was applied in the TOPALi v2.5 program package (1000 samplings).

## Results

### Gross and microscopic pathology

**Fig. 1:**
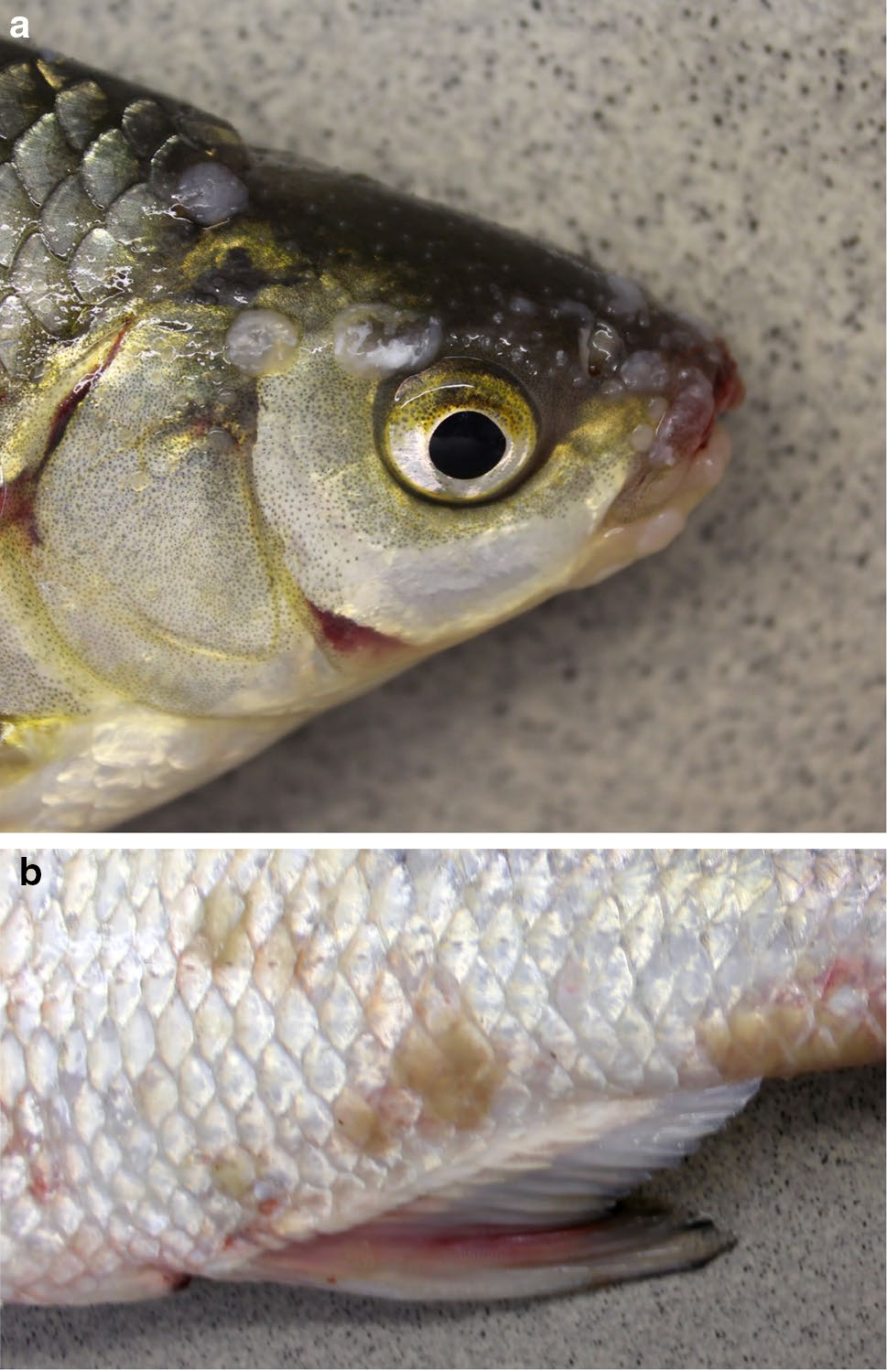
Photos displaying external gross pathology. (a) Roach infected with cyprinid herpesvirus 5 (CyHV-5) showing foci of epidermal hyperplasia. (b) Asp infected with CyHV-5 displaying foci of epidermal hyperplasia

**Fig. 2:**
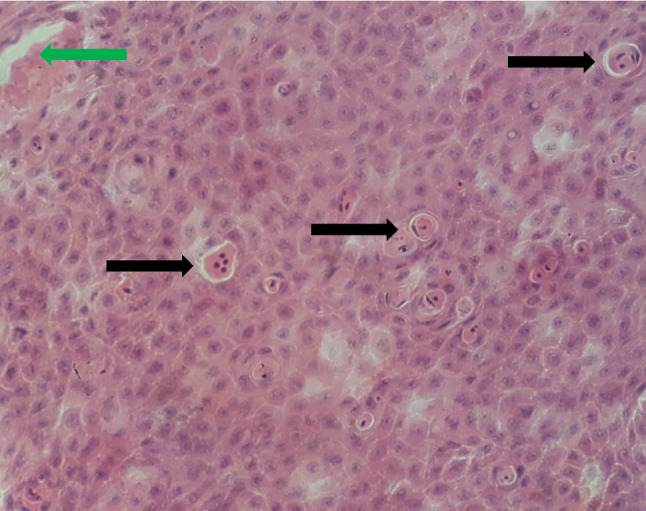
Histologic section of a carp-pox-like lesion showing benign epidermal hyperplasia. Several areas of apoptosis (black arrows) and some necrosis of the tumor cells (green arrow) could be seen in the hyperplastic tissue. Keratinization of the tissues was not observed. Intranuclear inclusions could not be distinguished in the lesions. 400x

Both the roach and the asp showed the typical external signs of CyHV-1 infection. Foci of hyperplastic epithelium were seen all over their bodies (Fig. 1). The carcass of the asp that was found dead in the fish trap was in slightly degraded condition; therefore, no histopathological examination was carried out. Histological examination of the carp pox-like lesion of the roach showed benign epidermal hyperplasia. Several foci of clusters of apoptotic cells and some necrosis of the tumor cells could be seen in the hyperplastic tissues. Keratinization of the tissues could not be observed, and intranuclear inclusions could not be distinguished in the lesions (Fig. 2). No histological lesions occurred in the gills, liver, spleen or heart of the diseased roach, except for the hepatic steatosis.

### Virus isolation

Virus isolation was attempted using the EPC and KF-1 cell lines with supernatants from of the homogenates of internal organs and of the hyperplastic tissues of the roach. No CPE typical for herpesviruses was observed in any of the inoculated cell cultures during the virus isolation attempts. After 14 days, a blind passage was carried out, and then in a fortnight a second blind passage was conducted. No CPE was observed in either the second or third passage, an attempt to detect viral DNA by PCR also failed in all cases.

### PCR assays and sequence analysis

In the DNA extracts of both the external lesions and of a mixture of internal organs of the roach and asp, the presence of alloherpesviral DNA was confirmed by PCR [12]. In these reactions, 300-bp-long DNA fragments from the DNA polymerase gene were amplified. The sequences of these fragments from the two hosts showed 99% nt sequence identity (4 nt differences causing 1 aa difference). BLASTx analysis of these partial DNA polymerase sequences suggested that the roach and asp carried a novel type of cyprinid herpesvirus, which we have named "cyprinid herpesvirus 5" (CyHV-5). Subsequently, portions of the MCP (574 bp) and the terminase (1008 bp) genes of CyHV-5 were amplified and sequenced. The two genes from the two different species shared 98% and 91% nt sequence identity resulting one and 10 aa differences, respectively. The sequences were deposited to the GenBank database under accession nos. MK507839-40, MK507844-45, and MK598760-61. The G + C content of the concatenated nucleotide sequences of CyHV-5 from the asp and roach was 51.43% and 52.73%, respectively. Attempts to amplify the helicase gene and the longer DNA polymerase fragment by the PCR procedures used previously for the characterization of CyHV-4 [10] failed to yield a product.

### Phylogenetic analysis

**Fig. 3:**
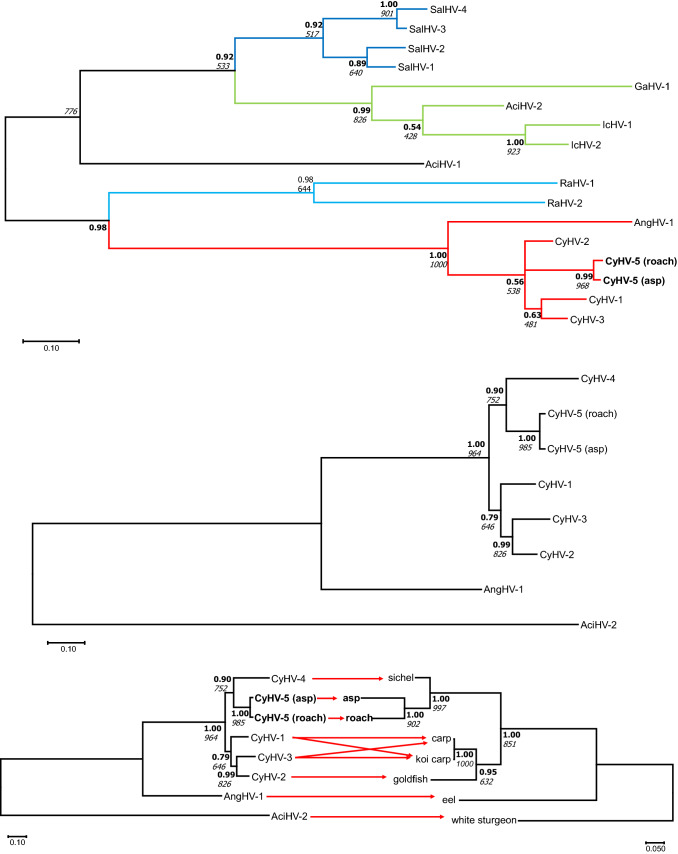
(a) Midpoint-rooted phylogenetic tree for alloherpesviruses. The analysis was based on Bayesian analysis (WAG amino acid [aa] substitution model) and the maximum-likelihood (ML) method (RTRev aa substitution model) of the partial DNA polymerase sequences (79 amino acids characters). Both methods produced the same tree topology. The main lineages (genera) within the family are indicated by different coloured lines on the tree. (b) Phylogeny reconstruction for the genus *Cyprinivirus* inferred by Bayesian analysis and ML (JTT aa model for both) using the concatenated amino acid sequences of DNA polymerase and terminase genes (411 amino acids). Both methods produced the same tree topology. AciHV-2 was selected as the outgroup. High statistical values confirm the topology of the trees. Posterior probability values are shown in bold characters, while the bootstrap values for ML are in italics. (c) Tanglegram of host-virus coevolution within the genus *Cyprinivirus*. Congruence was observed between the phylogenetic relationships among CyHVs after ML and Bayesian inference of the concatenated sequences of the DNA polymerase and terminase genes (right) and host species after ML and Bayesian inference of mitochondrial sequences (1138 nucleotides from the cytochrome-b gene) (left). Abbreviations: AciHV, acipenserid herpesvirus; AngHV, anguillid herpesvirus; CyHV, cyprinid herpesvirus; GaHV, gadid herpesvirus; IcHV, ictalurid herpesvirus; RaHV, ranid herpesvirus; SalHV, salmonid herpesvirus

Figure 3a shows the phylogenetic relationships within the family *Alloherpesviridae*, with CyHV-5 clearly clustering with members of the genus *Cyprinivirus*. The Bayesian and ML trees were both supported by high statistical values, however, polytomy was seen within the genus. The exact relationships could not be fully resolved within the genus using this short DNA polymerase sequence. The phylogenetic tree reconstruction (based on the concatenated sequences of the DNA polymerase and terminase genes) presented in Fig. 3b, illustrates the clear separation of CyHV-5 from the other CyHVs. Analysis based on longer sequences resolved the position of the novel AlloHV within the genus *Cyprinivirus* and showed it to be a sister species of CyHV-4. A tanglegram (Fig. 3c) suggested that the cyprinid herpesviruses mostly have coevolved with their hosts species. Table 2 shows a comparison of the concatenated nucleotide sequences (1883 bp in length) of CyHV-5 with CyHV-1, -2, -3, and -4. The sequence identity values fall in a narrow range (72-76%), which supports the assumption that the novel AlloHV is a member of a distinct virus species and not only a variant of a known CyHV.

**Table 2:** Comparison of the concatenated nt sequences (DNA polymerase, major capsid protein and terminase) of the different CyHVs

	CyHV-1	CyHV-2	CyHV-3	CyHV-4	CyHV-5
CyHV-1	-				
CyHV-2	73%	-			
CyHV-3	75%	74%	-		
CyHV-4	72%	73%	74%	-	
CyHV-5	76%	76%	76%	73%	-

The values in the body of the table are the nucleotide sequence identity values for the corresponding gene regions

## Discussion

Cyprinid fish are economically very important, and hence the global spread and severe negative impact of herpesviruses on wild and cultured populations of cyprinid fish species have been well documented, and studies are ongoing [16]. In this study, we examined a wild roach and an asp (both belong to the family Cyprinidae) displaying the typical signs of carp pox disease, and we provided the first molecular data about the genome of a novel AlloHV detected by PCR in these specimens. After the first report of CyHV-3 [19], there was a long pause in the discovery of novel cyprinid herpesviruses. Fifteen years had passed when a previously unknown AlloHV (CyHV-4) from sichel was reported in Lake Balaton [10]. Now, four years later, in the same lake, based on a partial genome sequence analysis of the detected AlloHV, we report the presence of a putatively novel CyHV causing carp pox-like disease.

An analysis of the partial DNA polymerase, major capsid protein, and terminase gene sequences showed that the viruses detected in wild roach and asp from Lake Balaton were almost identical, with 99, 99,5 and 97% aa sequence identity, respectively, suggesting that they belong to the same virus species. There are no species demarcation criteria for herpesviruses based on sequence data only. A herpesvirus may be classified as a member of a distinct species if it has distinct biological or epidemiological characteristics and a distinct genome sequence that represents an independent replicating lineage [26]. For genetic analysis, the *Herpesvirales* Study Group of the ICTV has not mandated that complete genome sequences be determined, has not set a dependence on any particular gene or genes (nor required that the sequence of any particular gene be complete), and has not specified genetic distance thresholds for differentiating taxa. The rule of thumb applied in previous proposals was that the evolutionary distance between the virus to be classified and its closest classified relative should be more than that between the most closely related viruses that have already been included in the family *Herpesviridae* (https://talk.ictvonline.org/files/ictv_official_taxonomy_updates_since_the_8th_report/m/animal-dna-viruses-and-retroviruses/8056). Our phylogenetic analysis showed that the viruses detected in roach and asp were clearly clustered with members of the genus *Cyprinivirus* within the family *Alloherpesviridae*. In addition, phylogenetic reconstruction inferred by maximum-likelihood and Bayesian analysis using the concatenated amino acid sequences of the DNA polymerase and terminase genes revealed that this virus should be regarded as a member of a new species in the genus *Cyprinivirus*, closely related to CyHV-4. A comparison of the concatenated nt sequences of the different CyHVs (Table 2) also supports the establishment of a new virus species for these viruses. It could be seen that the nt sequence identity values among the viruses (members of accepted species and new ones as well) range in a narrow interval (72-76%).

Most herpesviruses have a restricted host range in which a productive infection can be established [5]. The successful replication of a viral agent in a host is a complex process that consists of a number of interactions, most of them related to the coevolution of pathogen and host. An applied co-phylogenetic analysis based on the concatenated sequences of the viruses and cytochrome b sequences of the hosts supported the hypothesis of co-phylogenetic descent of the main lineages of CyHVs and their piscine host (Fig. 3c). This coevolution often leads to a species specificity of the virus and can make interspecies transmission difficult. Therefore, natural host range switches by viruses are rare events. However, when they occur, the results can become severe because the viruses may then spread widely through previously non-adapted and therefore immunologically naïve host populations [3].

The close genetic relationship of the viruses from roach and asp were evident from the high nt and deduced aa sequence identities of the conserved DNA polymerase, MCP and terminase genes. Most probably, these viruses should be considered genetic variants of the same virus species. It is impossible to say whether the roach or asp was the original host of the virus and which way the host jump occurred. However, the host switch from roach to asp has a higher probability, since asp prey on roach. Previous host switches among cyprinid herpesviruses must have occurred, since CyHVs can infect fishes of different superorders (Elopomorpha, Ostariophysi).

It was mentioned in the introduction that signs of carp pox disease supposedly caused by CyHV-1 have been described in approximately 10 cyprinid fish species, but no molecular investigations have been carried out [7, 24]. Here, we provide some molecular evidence that a putatively novel virus species might cause similar signs in one of the hosts mentioned earlier (roach) and in a novel species (asp). These results highlight the need for screening and detailed molecular analysis of closely related host species showing signs of the carp pox disease.

In this study, we genetically characterized a putatively novel AlloHV from roach and asp suffering from the so-called carp pox disease. Given that he sequences of the CyHV-5 genes that were examined differ markedly from those of the four known cypriniviruses, we propose that the species designation "*Cyprinid herpesvirus 5*" be considered for approval by the ICTV. Future studies (isolation of the virus and subsequent controlled challenge studies) are needed to determine whether this novel AlloHV causes carp-pox-like disease.
